# Tackling Heterogeneous Light Detection and Ranging-Camera Alignment Challenges in Dynamic Environments: A Review for Object Detection

**DOI:** 10.3390/s24237855

**Published:** 2024-12-09

**Authors:** Yujing Wang, Abdul Hadi Abd Rahman, Fadilla ’Atyka Nor Rashid, Mohamad Khairulamirin Md Razali

**Affiliations:** 1 Faculty of Information Science and Technology, Universiti Kebangsaan Malaysia, Bangi 43600, Selangor, Malaysia; p131146@siswa.ukm.edu.my (Y.W.); p111390@siswa.ukm.edu.my (M.K.M.R.); 2 Faculty of Physics and Electrical and Electronic Engineering, Aba Teachers University, Wenchuan 623002, China; 3 Center for Artificial Intelligence Technology, Universiti Kebangsaan Malaysia, Bangi 43600, Selangor, Malaysia; fadilla@ukm.edu.my

**Keywords:** data representation, object detection, heterogeneous alignment, multimodal sensors

## Abstract

Object detection is an essential computer vision task that identifies and locates objects within images or videos and is crucial for applications such as autonomous driving, robotics, and augmented reality. Light Detection and Ranging (LiDAR) and camera sensors are widely used for reliable object detection. These sensors produce heterogeneous data due to differences in data format, spatial resolution, and environmental responsiveness. Existing review articles on object detection predominantly focus on the statistical analysis of fusion algorithms, often overlooking the complexities of aligning data from these distinct modalities, especially dynamic environment data alignment. This paper addresses the challenges of heterogeneous LiDAR-camera alignment in dynamic environments by surveying over 20 alignment methods for three-dimensional (3D) object detection, focusing on research published between 2019 and 2024. This study introduces the core concepts of multimodal 3D object detection, emphasizing the importance of integrating data from different sensor modalities for accurate object recognition in dynamic environments. The survey then delves into a detailed comparison of recent heterogeneous alignment methods, analyzing critical approaches found in the literature, and identifying their strengths and limitations. A classification of methods for aligning heterogeneous data in 3D object detection is presented. This paper also highlights the critical challenges in aligning multimodal data, including dynamic environments, sensor fusion, scalability, and real-time processing. These limitations are thoroughly discussed, and potential future research directions are proposed to address current gaps and advance the state-of-the-art. By summarizing the latest advancements and highlighting open challenges, this survey aims to stimulate further research and innovation in heterogeneous alignment methods for multimodal 3D object detection, thereby pushing the boundaries of what is currently achievable in this rapidly evolving domain.

## 1. Introduction

The development of autonomous driving, intelligent transport systems, and environmental sensing technology has received widespread attention and occupies a pivotal position [1,2,3,4,5]. The main task of perception is to accurately and precisely understand the complex environment surrounding a vehicle and minimize the potential risk of collision, which is the cornerstone of safe and efficient mobility [6,7]. Environmental perceptions and object detection are closely linked. In scenarios such as highway driving, three-dimensional (3D) object detection can accurately identify vehicles and obstacles at different distances, while in urban traffic, even in cluttered environments, 3D object detection can support the accurate detection of smaller objects such as bicycles and pedestrians. As a result, 3D object detection technology is becoming increasingly important. It has become a driving force for the development of autonomous driving and intelligent transportation systems to a higher stage.

Multisensor fusion integrating data from Light Detection and Ranging, or LiDAR, camera, and radar, plays a crucial role in overcoming the limitations of single-mode systems and ensuring robust object detection in diverse, dynamic environments [8,9,10]. For example, in autonomous driving, the fusion of LIDAR and a camera enables advanced functions such as lane detection in poorly lit conditions, while in dense urban environments, sudden changes in light or pedestrian occlusion also challenge existing single-mode systems.

### 1.1. Motivation

With the rapid development of autonomous driving and intelligent transportation systems, precise 3D object detection in dynamic environments has become one of the key technologies for ensuring safety and reliability. In the 3D object detection task, LiDAR and cameras, as complementary sensors, provide sparse and precise geometric information and rich semantic visual information, respectively, and their fusion helps improve the accuracy and robustness of target detection. However, achieving efficient cross-modal alignment and fusion is still challenging due to the significant differences in modal data characteristics, especially in dynamic scenes, where fast-moving targets, difficulties in temporal and spatial synchronization of modal data, and the exploitation of characteristics under noise interference limit the applicability of existing methods [11].

On the other hand, the complexity of objects in dynamic environments imposes higher requirements on multimodal data representation. In dynamic environments, object detection faces the following challenges: rapid target motion, occlusion, interaction, and environmental changes. Uncertainty in dynamic scenes makes multimodal heterogeneous alignment methods vital tools for coping with target detection accuracy and real-time performance. The difference between the sparseness of LiDAR point clouds and the denseness of camera images makes it particularly difficult to construct uniform and robust feature representations of dynamic scenes. Existing feature extraction and fusion methods may suffer from delays or information loss in rapidly changing scenes, thus affecting the real-time reliability of detection. Therefore, a systematic review of feature alignment and data representation methods for LiDAR cameras is important for promoting efficient 3D object detection in dynamic environments.

This review aims to comprehensively summarize existing research, explore the technical difficulties and solutions for LiDAR-camera alignment and fusion in dynamic environment detection, and identify the advantages and shortcomings of the current technologies. By systematically analyzing the current status and challenges of multimodal fusion in dynamic scenes, it is hoped to provide theoretical support and practical references for future research, promote the development of more robust and efficient 3D object detection methods, and lay the foundation for practical applications in the fields of autonomous driving and intelligent transportation.

### 1.2. Existing Surveys

Existing surveys focus on either camera, LiDAR, or multimodal object detection network methods. As shown in Table 1, most relevant studies have been conducted in the last few years. They cover multiple components of datasets, sensor technologies (e.g., LiDAR and cameras), deep learning techniques for feature extraction and object localization, data representation, and alignment methods for object detection networks. While they provide valuable insights into specific aspects, such as sensor modalities, LiDAR data representations, and general pipelines for 3D detection, they exhibit notable limitations. Specifically, they need comprehensive discussions on the intricate details of multimodal data representation and alignment, which are crucial for integrating heterogeneous sensor inputs.

The primary challenge lies in the differences between camera-recorded information, which projects the real world onto a two-dimensional (2D) plane, and point clouds, which directly store spatial geometric information. The main issue to overcome is multimodal fusion, followed by addressing the disparity in data representation. Point clouds are irregular, unordered, and continuous, whereas images are regular, orderly, and discrete. These fundamental differences pose obstacles in developing algorithms for processing point cloud and image data, including the need for calibration between the two systems. Overcoming these disparities is crucial for advancing the study of LiDAR and camera data fusion in perception systems [12,13,14]. The Convolutional Neural Network (CNN) method extracts and fuses features from LiDAR point clouds and camera images by stacking multiple convolutional layers to achieve data alignment. However, this can lead to issues of high computational complexity and limited real-time performance [15,16,17]. Furthermore, the aforementioned surveys need to address the challenges and methods of multimodal object detection in dynamic and complex environments, where real-time adaptability, occlusion handling, and sensor fusion robustness are critical. This gap underscores the need for a more detailed review of heterogeneous alignment in this area.

**Table 1:** Comparison of surveys on object detection. (√) indicates that the topic has been covered. (×) indicates that the topic was not covered. (⊙) indicates that the topic is partially covered.

Year	Surveys	Representation	Alignment	Datasets	Challenges	Main Topic
2019	[18]	⊙	×	√	×	Multimodal detection methods.
2020	[19]	⊙	×	√	×	LiDAR-based deep networks.
2021	[20]	√	×	⊙	√	LiDAR-based detection.
	[21]	×	×	√	×	Electric vehicles detection.
	[22]	⊙	×	√	√	LiDAR-based detection.
2022	[23]	⊙	×	√	√	Case study detection methods.
	[24]	×	×	√	×	LiDAR-based detection.
2023	[7]	√	⊙	√	√	Multimodal detection methods.
	[25]	√	⊙	√	√	Images-based detection.
	[6]	×	×	√	√	Multimodal detection methods.
2024	[26]	⊙	⊙	√	√	Multimodal detection methods.
	[27]	⊙	⊙	√	√	Multimodal detection methods.
This study		√	√	√	√	Representation and alignment.

### 1.3. Contributions

Current research on object detection primarily focuses on the statistical analysis of fusion algorithms, while in real-world dynamic scenarios, the detection performance of these algorithms depends on the unified representation and alignment of multimodal data.

This review focuses primarily on the statistical analysis of fusion algorithms in object detection, emphasizing the advancements in heterogeneous data processing and classification. It begins by summarizing key developments in the field and identifying primary studies. These studies are analyzed, including categorizing data representation and alignment methods. The review concludes with a discussion of the challenges and recommendations for future research. This paper addresses four research questions (RQ) left unresolved by previous reviews:

1. What are 3D object detection, related autonomous driving datasets, and their heterogeneous alignment?
2. How many studies on data representation and heterogeneous alignment methods for 3D object detection have been conducted between 2019 and 2024?
3. How do we categorize 3D object detection data representation and heterogeneous alignment methods?
4. What are the challenges, limitations, and recommendations for future research on 3D object detection?

In this paper, current articles on heterogeneous alignment methods for 3D object detection proposed in recent years are discussed. Firstly, the basic concepts of 3D object detection and the screening method are described. Secondly, the 3D object detection heterogeneous alignment methods are classified according to different characteristics. Finally, the shortcomings of current 3D object detection algorithms for heterogeneous data alignment are analyzed, and future development trends are predicted. The contributions of this study are as follows.

1. Provide an analytical comparison of 3D object detection and heterogeneous alignment, focusing on recent research articles published between 2019 and 2024.
2. Summarize the latest research trends and a method for classifying the alignment of heterogeneous data for 3D object detection.
3. Highlight critical challenges in the heterogeneous alignment of 3D object detection and potential avenues for future exploration in this domain.

### 1.4. Organization

**Figure 1:**
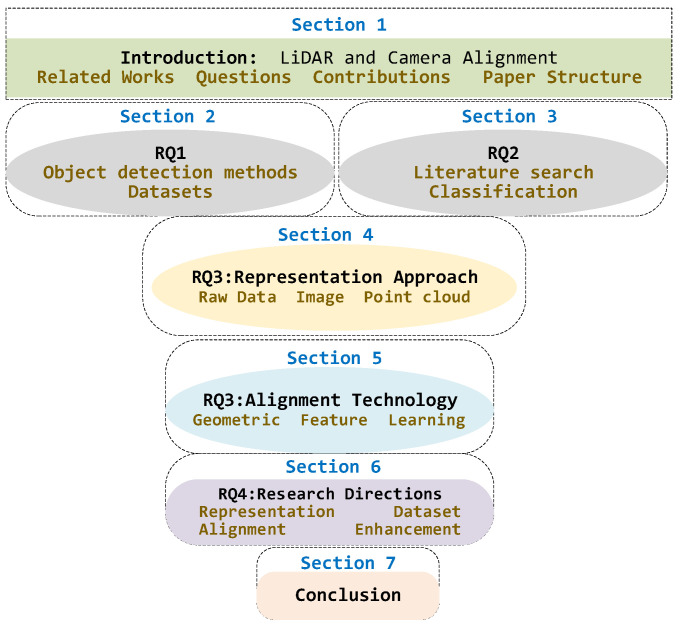
Structure and organization of this paper.

The structure of this paper is shown in Figure 1. Section 1 provides a complete introduction. Section 2 provides an overview of 3D object detection, covering the definition of object detection, sensor characteristics, various detection methods, and relevant datasets (RQ1). Section 3 outlines the steps for identifying significant studies between 2019 and 2024 (RQ2). Section 4 and Section 5 then present the representation methods and alignment strategies, including the heterogeneity of multimodal data in the 3D object detection addressed (RQ3). Section 6 summarizes the analytical discussion of this review and outlines the directions for future research (RQ4). Section 7 concludes this review.

## 2. Background

This section introduces the background of 3D object detection and common dataset indicators encompassing RQ1. It also highlights the rationale behind the focus of this review. Unlike previous works that primarily concentrated on single-modal or multimodal object detection methods, our review emphasizes the representation and alignment of multimodal data. This perspective addresses the foundational challenges of effectively integrating and utilizing multimodal information, thereby providing a more holistic understanding of the field.

### 2.1. Object Detection

#### 2.1.1. Sensory

Object detection aims to predict objects’ bounding boxes and properties, including their position, size, and category. Sensors can provide raw data for 3D object detection. Cameras and LiDAR sensors are the two most commonly used sensors. Cameras have advantages such as low cost, easy accessibility, and high frame rate, which are crucial for understanding semantics. The camera generates an image *I_cam_* ∈ *R^W^^×H^^×3,^* which can be used for feature extraction, where *W* and *H* are the width and height of the image, respectively. The captured image has rich texture and color characteristics. However, cameras have notable limitations: they capture only 2D appearance information and cannot directly provide the 3D structural details of a scene. While depth information can be inferred from images, it often involves significant errors. Furthermore, image-based detection is sensitive to lighting conditions and extreme weather, making nighttime detection considerably more challenging than daytime detection, which affects the robustness of object detection.

In contrast, a point cloud is data consisting of many points in the same spatial reference system, expressing the spatial distribution of objects. The point cloud can be represented as *I_point_* ∈ *R^N^^×3^*, and can be used for feature extraction in object detection. LiDAR sensors provide highly accurate, dense, and high-resolution point cloud data. However, LiDAR sensors are significantly more expensive than cameras, which limits their widespread adoption, particularly in cost-sensitive applications. Additionally, their resolution is lower than that of cameras, making the detection of small or distant objects challenging.

A comparison of cameras and LiDAR sensors highlights their complementary strengths and weaknesses. Cameras are well suited for capturing detailed appearance and semantic information, whereas LiDAR provides superior depth, accuracy, and robustness under varied lighting conditions. This comparison is crucial for understanding the trade-offs involved in sensor selection and motivates the exploration of multimodal approaches that integrate data from both types of sensors. By leveraging the complementary strengths of cameras and LiDAR, researchers have aimed to address the limitations of each individual sensor and enhance the performance of 3D object detection systems, especially in dynamic and complex environments.

#### 2.1.2. Camera-Based

In recent years, several well-established methods for 2D object detection have been developed for 3D object detection [28,29,30]. For example, image-based detection methods have a low cost [31,32]. Monocular cameras provide shape and texture in the form of pixels and therefore lack depth information [33,34,35]. Stereo cameras can provide accurate depth at the cost of complexity and cost increase [36,37]. Multiview cameras can generate depth maps covering different scene ranges than other cameras [38,39]. However, camera-based detection can be affected by unfavorable conditions, such as low light [40,41]. For the KITTI [42] dataset, the top monocular image-based method, DD3D [43], and the top stereo image-based method, LIGA-Stereo [44], achieved low reliability at 16.87% and 64.66% mean Average Precision (mAP), respectively. Further exploration is needed to achieve more reliable visual monitoring.

#### 2.1.3. LiDAR-Based

LiDAR provides robust 3D geometric information compared to camera images, which is critical for 3D object detection. Additionally, LiDAR sensors can better adapt to external factors, such as glare, resulting in more reliable detection under extreme lighting conditions. However, LiDAR-only algorithms have not been widely used in vision for the following reasons: (1) LiDAR is very expensive and bulky, especially compared to cameras [45]; (2) LiDAR has a minimal operating distance, and the point cloud away from LiDAR is very sparse [46]; and (3) LIDAR systems face significant challenges in extreme weather conditions such as heavy rain, as water droplets can disrupt the depth sensing of the LIDAR [47]. Moreover, the visibility of the camera image is reduced. Therefore, even with data fusion, extracting meaningful information under these conditions remains a challenge.

#### 2.1.4. Fusion-Based

Multimodal 3D object detection combines the advantages of multiple modalities by integrating various sensors to achieve better performance. Compared to unimodal detection, it can fully utilize depth information from point clouds and texture information from images. However, several challenges remain unsolved. Early pioneers in multimodal 3D object detection included MV3D [12]. They focused on combining data from two modalities for applications but overlooked the heterogeneous modal gap.

#### 2.1.5. Discussion and Analysis

Each sensor has unique characteristics, and their advantages sometimes complement each other. More methods are being developed to fuse networks of images and point clouds to achieve better performance than single-sensor methods in 3D object detection tasks. However, 3D object detection methods with fused networks must deal with heterogeneous data representations. The detection of point clouds and images requires specialized fusion mechanisms. 3D object detection methods typically utilize different projection views, such as bird’s-eye, cylindrical, and point views, to generate object predictions. Additionally, more research is now focusing on using modeling approaches for object prediction.

### 2.2. Datasets

#### 2.2.1. Definition and Comparative Analysis

Many driving datasets have been created in the past few years, facilitating research on 3D object detection by providing multimodal sensory data and 3D annotations. We present a survey of existing datasets (Table 2) and review the characteristics of popular datasets.

**Figure 2:**
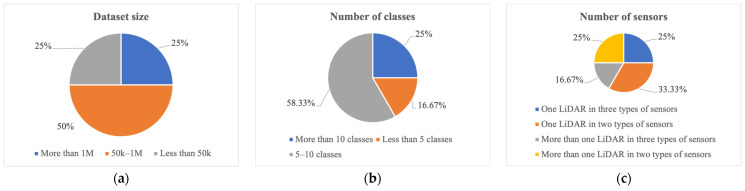
Frequency distribution of datasets based on (**a**) dataset size, (**b**) number of classes, and (**c**) number of sensors.

Existing popular datasets can be analyzed according to three groups. In the first group, the datasets are grouped by their size. In group 1, the sizes of the KITTI [48], KAIST [49], and Cirrus [56] datasets are less than 50 k. The second group classifies the datasets by class number. The class number of three datasets is more than 10. In the third group, datasets are categorized by the number of sensors. The numerator represents the LiDAR number, and the denominator expresses the number of sensors. The KITTI [48], KAIST [49], H3D [44], Argoverse [51], nuScenes [52], A*3D [54], and ONCE [57] datasets include only one LiDAR, whereas others use more than one LiDAR. The KITTI [48], Argoverse [51], A*3D [54], and ONCE [57] datasets include two sensor types, whereas the other datasets have three sensor types. The frequency distribution of the groupings is summarized in Figure 2.

#### 2.2.2. Discussion and Analysis

Autonomous driving datasets have different characteristics. The KITTI [48], Argoverse [51], A*3D [54], and Cirrus [56] datasets contain only driving data obtained during the daytime and in good weather. Recent datasets, such as KAIST [49], ApolloScap [43], H3D [44], Lyft Level 5 [50], nuScenes [52], Waymo [53], PandaSet [55], and ONCE [57], provide data captured at night or during rainy weather. Some datasets, like Argoverse [51], PandaSet [55], and nuScenes [52], offer finer object categories with more than 10 classes, while fewer than 10 categories are detected in two other datasets.

Precision and recall are the most commonly used metrics for object detection. Precision is the ratio of the number of instances correctly identified as positive samples by the model to the total number of instances correctly identified as positive samples. In contrast, recall is the ratio of the number of instances correctly identified as positive samples by the model to the total number of instances that are positive samples. The mAP and Normalized Detection Score (NDS) are also commonly used metrics for evaluating object detection tasks, measuring the average performance of the model across multiple categories. A higher mAP indicates better model performance on the detection task. The NDS considers the degree of overlap (IoU) between the object detection results and the ground truth. It weighs the recall and false alarm rates according to the size and distance of the target. Evaluation metrics may be adjusted, updated, or expanded as autonomous driving technologies and datasets evolve.

### 2.3. Heterogenous Alignment Discussion and Analysis

**Figure 3:**
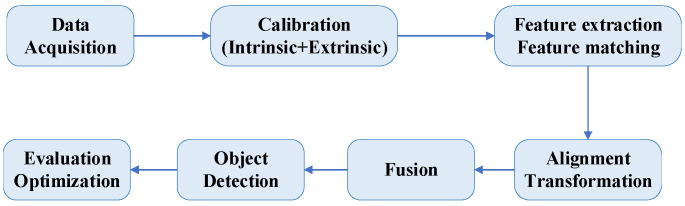
Magnetization frame of multimodal alignment and fusion.

In the context of 3D object detection for autonomous driving, heterogeneous alignment refers to the process of aligning and fusing data from different sensors. This alignment integrates the complementary characteristics of data from various sensors, enhancing object detection and recognition accuracy and robustness. By leveraging the strengths of each sensor type, heterogeneous alignment provides a more comprehensive understanding of the surrounding environment, which is crucial for the safe and effective operation of autonomous vehicles. The alignment is represented throughout the alignment and fusion process, as shown in Figure 3.

LiDAR and camera heterogeneous alignment aim to synchronize and integrate sensory input data from both LiDAR and camera systems to enhance the accuracy and robustness of 3D object detection. LiDAR sensors provide precise depth information and a detailed 3D point cloud representation of the environment, capturing the spatial structure of objects. In contrast, camera systems offer rich color and texture details, which are crucial for accurate object classification and identification. By aligning the data from these two heterogeneous sources, we can leverage the strengths of both modalities to achieve a more comprehensive understanding of the scene.

In dynamic autonomous driving environments, these sensors need to be effectively fused to compensate for the limitations of a single sensor and provide accurate environmental sensing. The alignment of heterogeneous sensor data plays a crucial role in system perception, decision-making, and planning. Although the data alignment of heterogeneous sensors can theoretically improve the performance of autonomous driving systems, it still faces many challenges in practice. In recent years, significant progress has been made in the application of deep learning and multimodal learning to image processing and point cloud processing. Data alignment from different sensors can be performed more efficiently using deep neural network models. However, in the face of dynamic and complex driving environments, overcoming the challenges of temporal synchronization, spatial alignment, and resolution differences in sensor data will be an important research topic in future autonomous driving.

## 3. Protocol and Strategies for Studies

This study aims to summarize the process of reviewing heterogeneous alignment and classifying LiDAR and camera fusion to answer RQ2.

### 3.1. Literature Search and Screening Strategies

In this study, we focused on the literature published between 2019 and 2024. A five-year time span is sufficient to analyze recent trends and draw conclusions. Databases searched for articles are WoS, SpringerLink, IEEE Xplore, ProQuest, ACM Digital Library, Scopus, Science Director, and Google Scholar. These databases were selected for research after considering expert opinions on the literature search strategies. The search keywords used were ‘machine learning’, ‘fusion or alignment’, ‘LiDAR’, ‘Camera’, and ‘object detection’, excluding terms such as ‘medicine’, ‘infrared’, ‘remote sensing’, and ‘mapping’. Boolean operators such as ‘AND’, ‘OR’, and ‘NOT’ were used to refine the search, with the ‘-’ operator in Google Scholar functioning as an equivalent to ‘NOT’.

The screening process for retrieving relevant articles consisted of four steps: removing duplicates, screening by title, screening by abstract, and screening for relevance to the research questions. The inclusion criteria for article screening are shown in Table 3. The screening results are presented in Table 4.

### 3.2. Classification and Analytical Framework

To provide a thorough overview of the current research on heterogeneous data alignment for multimodal 3D object detection, this study presents a systematic analysis of 21 representative papers published between 2019 and 2024. The analysis framework encompasses key dimensions, including country of origin, publication year, datasets utilized, methodological approaches, primary contributions, and the identified limitations in Table 5. The examined studies predominantly originate from China (17 papers, or 80.9%) [58,59,60,61,62,63,64,65,66,67,68,69,70,71,72,73,74], with additional contributions from the United Kingdom [75], Germany [76], the United States [77], and Brazil [78]. The KITTI dataset is the most frequently employed benchmark (11 papers, or 52.3%) [58,59,61,63,64,66,67,68,75,78], supplemented by other datasets such as CADC, SUN-RGB [65], FlyingThings3D [73], Waymo [53], and nuScenes [52,71,72,73,74,77]. This highlights the importance of the KITTI dataset, followed by the nuScenes dataset.

## 4. Heterogeneous Data Representation Approaches

RQ3 is discussed in two parts: 3D object detection data representation and heterogeneous alignment methods. In this section, data representation approaches are examined.

### 4.1. Categorization Summary

The previous subsection analyzed the literature from 2019 to 2024, focusing on multimodal data, primarily point clouds, and images collected by LiDAR and cameras. These data types are not uniformly represented. This subsection expands the analysis to the past decade, summarizing the fact that heterogeneous data representations in 3D object detection can be categorized into primitive and projective representations. This categorization is based on a literature review from the last five years. Table 6 provides a detailed summary of these findings.

The data representations from LiDAR and cameras used in 3D object detection vary, each with advantages and disadvantages. These are generally categorized into raw data representation, project representation, and bird’s eye view (BEV) representation.

Raw data representation includes raw image data and raw point cloud data. Raw image data is a two-dimensional representation of an image that provides much information and improves detection accuracy, but requires a lot of computation and long inference time [45,67,71,79,80,81,82,83,84,85,86,87,88,89,90,91,92,93,94,95,96,97,98,99,100,101,102,103,104,105,106,107,108,109,110,111,112]. Raw point cloud data are three-dimensional data directly generated by LiDAR sensors, which are usually rich in spatial information and highly accurate in practical applications [81,82,84,85,86,89,90,91,94,99,100,102,106,107,108].

Project representations include pseudo-point cloud 3D representations generated from images, which have the advantages of low computational effort and fast processing speed, but contain less information and lose geometric details in the conversion process [96,113]. The BEV representation consists of BEV feature data and BEV map data [114,115,116,117]. BEV feature data are 2D representations derived from an image suitable for feature extraction and global analysis.

A voxel is a typical representation derived from point clouds, characterized by its ability to discretize the 3D space into uniform grids, allowing for efficient spatial indexing and processing of large-scale data [71,83,92,94,95,97,98,100,101,103,104,105,109,110,111,112,113,114,115]. Front (range) view data are a 2D representation generated from a point cloud that simplifies the computation [87,88]. BEV map data are a 2D representation derived from point clouds, which can provide more intuitive spatial information for vehicle path planning and environment understanding [12,45,96,107,117,118,119].

### 4.2. Discussion and Analysis

Different data representations, such as images and point clouds, exhibit distinct strengths and weaknesses that influence their suitability for various application scenarios. Images typically contain rich information, such as color and texture, which contributes to improved accuracy in object detection. However, the computational costs and inference times associated with image processing are often significant. In contrast, point clouds generated by sensors like LiDAR are computationally efficient and offer faster processing speeds, but they generally lack the detailed contextual and semantic information found in images. Additionally, point clouds may suffer from geometric information loss during processing. While BEV representations derived from point clouds enhance real-time performance, they introduce projection errors, which may reduce accuracy, especially for detecting small or distant objects. These trade-offs highlight the importance of selecting appropriate data representations based on the specific demands of multimodal object detection in dynamic environments.

**Figure 4:**
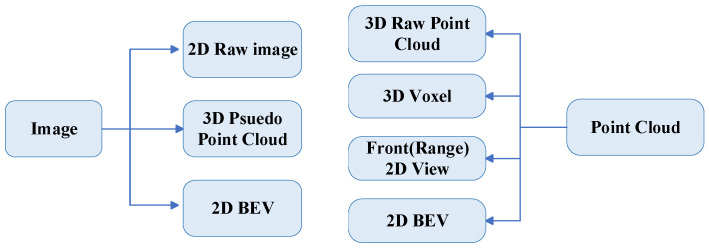
Magnetization Frame of Multimodal alignment and fusion.

Since input data for multimodal fusion are often heterogeneous, constructing correspondences between the features of different modalities is critical. Researchers have proposed various alignment methods to address this challenge, enabling effective data integration from different sensors. For instance, a common approach is to represent LiDAR point clouds and camera images in a shared feature space, as illustrated in Figure 4. By analyzing the strengths and weaknesses of these data representations, we aim to facilitate advancements in solving the complex problem of multimodal object detection in dynamic environments.

## 5. Heterogeneous Alignment Techniques

**Figure 5:**
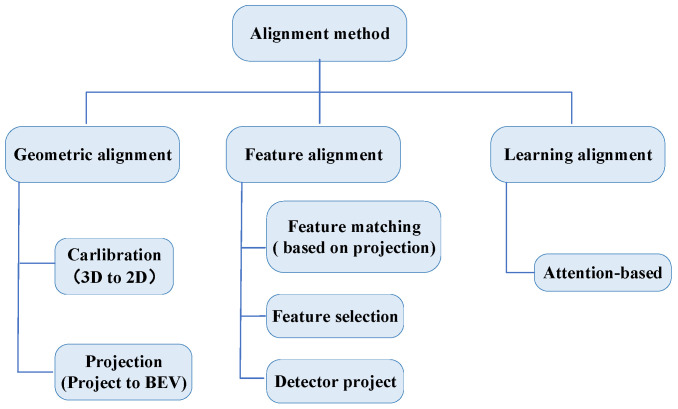
Summary of alignment approaches.

To answer RQ3, this section categorizes the alignment methods from reviewed papers into three types: (1) geometric alignment, (2) project alignment, and (3) learning alignment. This paper identified several studies that incorporate similar approaches. Analyzed from Table 5, three papers implemented the geometric and project alignment approach. The primary studies implemented 3D object detection through feature matching [58,60,67,68,69], achieved feature alignment of both sensors by feature selection [66,75], mapped 3D features into 2D features to achieve feature alignment [59,78], and directly fused the data generated by LiDAR and camera sensors after extracting features [64]. Pang et al. [73] directly processes the 3D detector head as a 2D detector head. Finally, other works have used an attention mechanism to achieve multimodal heterogeneous data alignment [61,63,65]. Figure 5 presents a summary of the alignment approach.

The geometric alignment method has high computational efficiency and good real-time performance; however, its accuracy is limited, and its ability to adapt to environmental changes is poor. The project alignment method can obtain higher accuracy and better adapt to environmental changes but requires a large amount of a priori knowledge and computational resources. The learning alignment method combines the advantages of project alignment and model alignment, which can trade-off accuracy and efficiency, but increases the algorithm’s complexity and the parameter’s difficulty.

### 5.1. Geometric Alignment

Geometric alignment methods can be roughly divided into Calibration and Projection.

Calibration: This is a crucial preprocessing step in multimodal heterogeneous data processing using a projection matrix that includes intrinsic and extrinsic parameters to project 3D world coordinates to 2D image coordinates [67,68], especially when combining 3D data with 2D data. It is important for multimodal data fusion to ensure the spatial consistency of different modal data (e.g., RGB images and LiDAR point cloud data) through calibration [59,78].

Projection: This is another preprocessing step for multimodal heterogeneous data. When 3D information needs to be recovered from a 2D image, points in the 2D image can be inversely mapped into the 3D space using the camera’s internal and external references, called a pseudo-point cloud [58]. This typically requires additional depth information or assumptions, such as the use of depth images or stereo vision, to recover 3D points. Alternativey projecting heterogeneous sensor data into a unified geographic coordinate system and achieving alignment through local feature matching, e.g., a uniform project into the BEV space [64], which is more efficient but less resilient to large-scale environmental changes.

### 5.2. Feature Alignment

Projection alignment after feature extraction is known as the feature alignment. It generally performs better for detection than for data alignment before feature extraction. Feature alignment can be divided into three categories based on specific methods.

Feature matching: Ouyang et al. [60] achieved multimodal data alignment by projecting a LIDAR point cloud onto a 2D image plane, generating a depth range diagram (DRD), and then combining these 2D features with camera images. Specifically, it first generates DRD maps from the sparse LIDAR point cloud using an up-sampling algorithm and then fuses the DRD maps with the image to enhance the accuracy of object detection. Wang et al. [69] fuses the semantic features of view voxels with the geometric features of the point cloud by projecting each point cloud onto an image feature map. In this way, PVFusion can better utilize the semantic information of the image, thus improving the detection accuracy of small objects (e.g., pedestrians) in sparse point clouds.

Feature selection: Features are fused by generating 3D and 2D regions of interest (RoIs) in point clouds and images, respectively, and then aligning these two RoIs. Specifically, RoIFusion generates 3D RoIs in the point cloud and corresponding 2D RoIs in the image, extracts geometric and texture features through the 3D RoIs pooling layer and 2D RoIs pooling layer, and finally fuses these features for 3D object detection. This approach effectively reduces the computational cost and viewpoint misalignment problems and improves the detection performance [71].

Detector project: Significantly improves the accuracy and real-time performance of 3D object detection by introducing a lightweight 3D-Q-2D image detector that combines 3D detection of candidate regions with image feature extraction, resulting in efficient alignment and fusion of point clouds and images [73].

### 5.3. Learning Alignment

Learning alignment has primarily focused on attention-based methods. Many researchers have leveraged deep learning methods for feature alignment and enhancing model alignment without sacrificing semantic information, mainly using attention [71,102,103,104,105]. AutoAlign [71] converts voxels into a query q and camera features into a key k and a value v. For each voxel cell, an inner product is computed between the query and the key, generating a correlation matrix between the voxel and all its corresponding camera features. This is followed by normalization, where the value *v*, containing the camera information, is weighted. To reduce the computational effort, Chen et al. [96] drew inspiration from deformable DETR [110] and proposed a cross-alignment algorithm called DeformCAFA, which employs a deformable cross-attention mechanism. In this method, the query *q* and key *k* are similar to AutoAlign, but the value *v* is modified. A projection matrix is used to query the image features corresponding to voxel features, and offsets are learned by an MLP, extracting the image features associated with these offsets as the value *v*. This enables each voxel to perceive the entire image, thereby facilitating feature alignment between the two modalities. Several studies have proposed projection and deep attention mechanisms for multimodal heterogeneous data alignment that effectively learn the significance of different modal features and adaptively fuse features based on a depth threshold [72,73,74,114]. The Graph Feature Alignment (GFA) module and Self-Attention Feature Alignment (SAFA) module introduced by Song et al. [73] improve the alignment accuracy of point cloud and image features by leveraging neighborhood relationships and attention mechanisms, ultimately boosting detection performance. Extensive experiments on the KITTI and nuScenes datasets demonstrate that GraphAlign enhances detection accuracy, particularly for small objects at long distances, in high-precision scenarios.

### 5.4. Discussion and Analysis

Multimodal data representations for object detection are categorized into raw and projected representations. Projected representation transforms data to enhance alignment and performance, particularly in BEV-based detectors, whereas raw representation retains the original data to preserve maximal information. Ongoing developments in multimodal representation suggest future advancements toward more efficient representations. However, heterogeneous sensor fusion remains an active research field, with new methods and algorithms continually emerging. In practical applications, it is essential to consider various factors, such as specific scenarios, required accuracy, and computational resources, when choosing the appropriate alignment method. For example, increasing the weight of lidar to achieve good detection in dynamic nighttime driving environments is a solution to this challenge.

The alignment of multimodal heterogeneous data is essential in the process of data fusion. The goal of data alignment is to effectively fuse heterogeneous data from different sensors and different modalities so that they have good correspondence in the same representation space and provide a unified multimodal input for subsequent sensing and prediction tasks. For heterogeneous data modalities, such as images and point clouds, it is difficult to fuse them directly due to the huge differences in their original representations. Therefore, it is necessary to map them to an intermediate representation space first, so that different modal data have similar representation forms in this space. The fusion of heterogeneous data into a unified representation space involves cross-modal data alignment and fusion. Common practices include using autoencoders to encode the data into the latent space, and using projection methods to map the data to the common space.

This review also summarizes the model alignment methods, and the common methods include the attention mechanism and the deformable attention mechanism. After data fusion, a unified multimodal representation not only facilitates complementary and synergistic information from different modalities, but also facilitates the application of machine learning models for subsequent perceptual inference tasks. Therefore, the core idea of the multimodal heterogeneous data alignment method is to fuse different modal data to generate a unified multimodal representation to achieve efficient fusion and utilization of inter-modal information. This is the basis and key to multimodal perception.

## 6. Challenges and Future Directions

**Figure 6:**
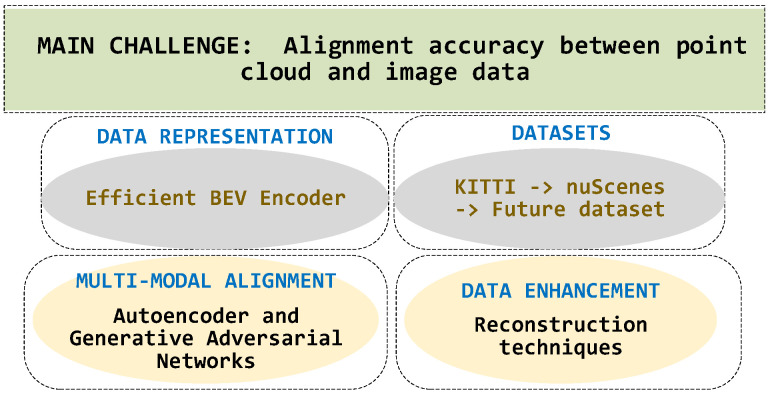
Structure and keywords of the challenges and research directions.

Despite many alignment techniques, the fusion of image and point cloud data in autonomous driving scenarios still faces challenges such as accuracy, robustness, and real-time processing requirements. Currently, improving the alignment accuracy between point cloud and image data is one of the main focuses of multimodal object detection research, and it is also the main challenge and limitation that the field is currently facing, and it still needs to fully mature. This section explores the current difficulties and emerging trends in the field of multimodal 3D object detection alignment techniques. The structure of future research directions follows the framework illustrated in Figure 6.

### 6.1. Data Representation

The heterogeneous nature of data acquired by different sensors can lead to problems, such as information desynchronization in data representation. Some recent studies by Liang et al. [114] and Liu et al. [115] attempted to use BEV representation to unify different modalities, which provides a new perspective to solve this problem and is worth further exploration.

The BEV representation can naturally fuse data from different sensors by projecting 3D data onto a horizontal plane and presenting scene information in a top-down view. Compared with the direct fusion of point cloud and image, BEV indicates that while preserving the rich semantic information of the camera, it also contains precise depth information of the point cloud, which is expected to resolve the differences between heterogeneous modalities better. Some recent works, such as BEVFusion and BEVFormer [120], are based on this idea and have achieved good results. However, there are some potential limitations of the BEV representation, such as the possibility of introducing additional errors in the projection process and the ability to model objects perpendicular to the ground is somewhat affected. Therefore, designing an efficient BEV encoder, solving the occlusion problem, and combining it with the original sensor data are all worthy of attention. More work is still needed to fully explore the potential of BEV representation to address challenges, such as projection error and occlusion. In addition, it can be combined with other techniques to obtain a more robust and accurate multimodal 3D object detection system.

### 6.2. Datasets

Most of the literature analyzed achieved better recognition results using KITTI datasets. However, KITTI datasets do not include data from outdoor scenes, such as nighttime. nuScenes datasets have attracted much attention due to their multimodal characteristics and wide coverage, which is expected to solve the challenge of the limited field of view of the current publicly available datasets in complex environments. In addition, traditional datasets are often limited to specific sensor types, such as a single camera or LIDAR, resulting in blind spots in complex scenes. By contrast, nuScenes integrates data from multiple sensors, including cameras, LiDAR, and radar, thereby providing a valuable resource for studying multimodal fusion perception.

Although nuScenes provides rich multimodal data, object detection still has some deficiencies. A complex urban scene and multiple object classes make detection more difficult. The calibration error and time delay between different sensor data also challenge data processing. Therefore, the focus of current research is on how to make full use of the advantages of multimodal data to improve the accuracy and robustness of object detection. Several studies have found that existing datasets still have some deficiencies in the object detection task. Some researchers have also pointed out that the poor quality of annotation and inconsistent format of annotation have brought some obstacles to the utilization of the dataset.

The development of future datasets needs to focus on the following aspects: (1) constructing datasets containing more scale variations to promote the performance of detection algorithms at different scales; (2) expanding the types of scenes for data collection, such as increasing the number of indoor, confined space, and other complex environments; (3) improving the quality of the annotation and adopting semiautomatic or automated annotation to reduce the error of manual annotation; (4) unifying the annotation format to facilitate the fusion of different datasets; and (5) exploring the use of fewer samples or unlabeled data to reduce the cost of labeling. Improving the multimodal sensing dataset will provide a more solid data foundation for the fields of automatic driving and robotics.

### 6.3. Multimodal Alignment

Although existing studies have attempted to encode sparse 3D representations into 2D representations [87,88], a large amount of information is often lost during the encoding process. This information loss not only affects the final perceptual accuracy but also increases the difficulty of subsequent processing. Therefore, encoding 3D features efficiently, reducing information loss, and achieving effective alignment of multimodal data becomes the key to improving the performance of multimodal perception.

Advanced coding techniques such as Autoencoder and Generative Adversarial Networks (GAN) can play an important role in deep learning to solve this challenge. Through unsupervised learning, the Autoencoder can learn the intrinsic feature representation of the data, encode high-dimensional sparse data into low-dimensional compact representations, and reconstruct the original data approximately when decoding. GAN learns the data distribution through adversarial training between generators and discriminators and generates synthetic data that are highly similar to the actual data, thus achieving efficient encoding and decoding.

By combining techniques such as autoencoders and GANs, researchers can develop more compact 3D representations that accurately align multimodal data while reducing information loss. For example, 3D point cloud data are encoded into a low-dimensional potential space, and then a reconstruction that is highly consistent with the original data is generated by a decoding network, thus preserving the key feature information. In addition, the GAN-based coding method can also generate more detailed and smooth correspondences for mapping between different modal data to improve the quality of multimodal fusion. In conclusion, the innovative application of efficient coding technology will promote the development of multimodal sensing algorithms and open new possibilities for autonomous driving in complex environments.

### 6.4. Data Enhancement

Data enhancement techniques are mainly applied to single-modal scenarios, with less consideration for multimodal situations [121,122,123,124,125,126]. Traditional data enhancement tools, such as rotation, translation, and scaling, mainly operate on a single data modality. It is difficult to capture and enhance the intrinsic connections between multiple modalities. Therefore, effectively enhancing multimodal data while preserving cross-modal correlation has become a key challenge in improving the performance of multimodal algorithms.

In response to the above challenges, some research works have attempted cross-modal data enhancement methods. For example, Wang et al. [106] proposed Point Enhancement, which is a more complex cross-modal data enhancement scheme for generating enhanced point cloud and image data pairs by adding additional mask annotations to image branches. However, this method requires additional manual labeling and is sensitive to noise, which still has some limitations. Therefore, better solutions are urgently needed to address cross-modal data enhancement’s synchronization and alignment challenges.

Reconstruction techniques may provide an effective way to solve this problem. By transforming heterogeneous multimodal data into a unified representation, the linkages between different modalities can be fully preserved. Based on this unified representation, we can perform data augmentation operations in the latent space to generate new representations, which can then be decoded into augmented multimodal data. This approach not only avoids the need for manual annotation but also effectively eliminates the influence of noise, thus achieving high quality cross-modal data enhancement. In the future, the integration of reconstruction technology and other machine learning models will provide better solutions for multimodal data enhancement and promote the development of multimodal perception algorithms.

## 7. Conclusions

This paper has provided a comprehensive examination of heterogeneous LiDAR-camera alignment methods in the context of multimodal 3D object detection. We explored the motivation and background of the field by presenting the existing datasets and evaluation metrics. In addition, we analyzed various datasets and evaluation metrics and compared the alignment and representation methods. Additionally, we presented a novel classification for data representation and feature alignment. We also discussed the advantages and disadvantages of different approaches. We provided an overview of recent trends, challenges, and future research directions. To overcome the deficiency of the traditional calibration method, we suggest a new alignment fusion method that maps the point cloud neighborhoods to the image neighborhoods by projection and then uses the self-attention mechanism to enhance the weights of essential relationships in the fused features.

It is important to acknowledge the limitations of this review, particularly regarding its scope. This review primarily addresses alignment methods without extensively exploring other critical aspects of object detection, such as data augmentation strategies, real-time processing capabilities, and the impact of emerging technologies like deep learning. Future reviews should consider a broader temporal scope to enhance our understanding of heterogeneous alignment methods and their applications in object detection. It would be beneficial to include discussions on the integration of novel machine learning techniques, implications of multimodal data processing on performance, and case studies from various domains, such as autonomous driving and robotics.

## Figures and Tables

**Table 2:** Summary of driving datasets for 3D object detection.

Datasets	Year	Dataset Size	Number of Classes	Number of Sensors	LiDAR Sensor	Camera Sensor	Sensor Types	LiDAR	Image	Classes	Locations
KITTI [48]	2012	<50 k	5–10	1/3	1	2	3	15 k	15 k	8	Germany
KAIST [49]	2018	<50 k	<5	1/2	1	2	2	8.9 k	8.9 k	3	Korea
ApolloScape [43]	2018	50 k–1 M	5–10	2/3	2	2	3	20 k	144 k	6	China
H3D [44]	2019	50 k–1 M	5–10	1/3	1	2	3	27 k	83 k	8	United States
Lyft L5 [50]	2019	50 k–1 M	5–10	2/2	2	6	2	46 k	323 k	9	United States
Argoverse [51]	2019	50 k–1 M	>10	1/2	1	7	2	44 k	490 k	15	United States
nuScenes [52]	2019	>1 M	>10	1/3	1	6	3	400 k	1.4 M	23	Singapore and United States
Waymo [53]	2019	>1 M	<5	5/2	5	5	2	230 k	1 M	4	United States
A*3D [54]	2019	50 k–1 M	5–10	1/2	1	6	2	39 k	39 k	7	Singapore
PandaSet [55]	2020	50 k–1 M	>10	2/3	2	6	3	8.2 k	49 k	28	United States
Cirrus [56]	2021	<50 k	5–10	2/2	2	1	2	6.2 k	6.2 k	8	China
NCE [57]	2021	>1 M	5–10	1/2	1	7	2	1 M	7 M	5	United States

**Table 3:** Inclusion and exclusion criteria for article screening.

Inclusion Criteria	Exclusion Criteria
Refer to LiDAR-camera representation and alignment	Refer to a point cloud representation only or others
A Machine Learning approach is followed	Not based on Machine Learning methods
Published in journal articles or conference papers	Published in elsewhere
Published between 2019 and 2024	Focus on mapping as the main topic

**Table 4:** Overview of the systematic screening.

Source	Initially Retrieved	After Removing Duplication	After First Screening	After Second Screening	After Third Screening
WoS	23	8	5	4	3
SpringerLink	15	13	4	1	0
IEEE Xplore	27	6	4	2	2
ProQuest	119	114	29	10	3
ACM Digital Library	11	11	3	1	1
Scopus	100	100	52	17	9
Science Director	11	11	6	2	2
Google Scholar	108	5	3	2	1

**Table 5:** Summary of contribution, applicability, and limitations of the primary studies.

Author	Country and Year	Dataset	Contribution	Applicability/Strength	Limitations/Trends
[58]	China 2022	KITTI	Image fusion with point cloud projection features.	Real-time detection.	Computation cost.
[59]	China 2021	KITTI	Sparse point cloud and image feature fusion.	Real-time detection.	Computation cost.
[60]	China 2022	KITTI: local dataset	Bilateral Filtering and Delaunay Point Cloud Densification	Dynamic environment.	Low accuracy of small objects.
[61]	China 2020	KITTI	Multilevel network of attentional mechanisms.	Dynamic environment.	Low accuracy of small objects.
[62]	China 2019	Real data	FL and YOLO CNN.	Diverse environment.	Safety.
[63]	China 2022	KITTI	2D feature extraction.	Occlusion environment.	Low accuracy of small objects.
[64]	China 2022	KITTI	Multiview and BEV feature.	Small-object detection.	Computation cost.
[65]	China 2021	KITTI; SUN-RGB	Multilevel network of attentional mechanisms.	Occlusion environment.	Computation cost.
[66]	China 2022	KITTI	Deep attention mechanism.	Occlusion environment.	Computation cost.
[67]	China 2023	KITTI	Modality adaptation.	Across different scenes	lightweight models
[68]	China 2022	KITTI	The multiscale feature aggregation module.	Across different scenes.	Real-time.
[69]	China 2023	KITTI	To improve the weight of image information.	Improve the weight of image information.	Diverse environment.
[70]	China 2022	Waymo	Enhancing sparse LiDAR data.	Various lighting conditions.	Computational complexity.
[71]	China 2023	KITTI; nuScenes	Pixel-level Cross-Attention Feature Alignment (CAFA).	Small objects.	Computational costs.
[72]	China 2023	KITTI; nuScenes	Graph Feature Alignment (GFA).	Small objects.	Real-time.
[73]	China 2022	KITTI; nuScenes	Self-Attention Feature Alignment (SAFA) modules.	Computational complexity.	Diverse conditions.
[74]	China 2024	nuScenes	Graph matching to resolve global misalignment.	Small-object detection.	Dynamic environment.
[75]	UK 2021	KITTI	Region of Interest deep learning	Low computation cost.	Low accuracy of small objects.
[76]	Germany 2020	KITTI; FlyingThings3D	Multiscale.	Dynamic environment.	Computation cost.
[77]	USA 2022	KITTI; nuScenes	Light weight.	Real-time detection.	Low accuracy of small objects.
[78]	Brazil 2020	KITTI	Late fusion.	Occlusion environment.	Computation cost.

**Table 6:** Summary of data representations for object detection.

References	Input Data	Dimension	Source
[45,67,71,79,80,81,82,83,84,85,86,87,88,89,90,91,92,93,94,95,96,97,98,99,100,101,102,103,104,105,106,107,108,109,110,111,112]	Raw Image	2D	Image
[81,82,84,85,86,89,90,91,94,99,100,102,106,107,108]	Raw Point Cloud	3D	Point Cloud
[96,113]	Pseudo-Point Cloud	3D	Image
[87,88]	Front (Range) View	2D	Point Cloud
[67,83,92,94,95,97,98,100,101,103,104,105,109,110,111,112,113,114,115]	Voxel	3D	Point Cloud
[114,115,116,117]	BEV Feature	2D	Image
[12,45,96,107,117,118,119]	BEV Map	2D	Point Cloud

## Data Availability

No new data were created or analyzed in this study. Data sharing is not applicable in this article.
